# Zwanzigjähriges Jubiläum der Interdisziplinären Arbeitsgemeinschaft für Klinische Hämotherapie: Ein Grund zum Feiern!

**DOI:** 10.1007/s00101-022-01105-y

**Published:** 2022-02-25

**Authors:** Thomas Frietsch

**Affiliations:** Interdisziplinäre Arbeitsgemeinschaft für Klinische Hämotherapie, An den Lahnbergen, 35043 Marburg, Deutschland

Transfusionsbeauftragte, Transfusionsverantwortliche und Qualitätsbeauftragte Hämotherapie als wichtige klinische Funktionsträger sind in ihren Einrichtungen für die korrekte Ausführung der Hämotherapie, also der Therapie mit zellulärem Blut und aus Blutplasma hergestellten Blutprodukten, zuständig. Die besondere Rolle dieser Therapie wird deutlich, wenn man sich die jährliche Häufigkeit der therapeutischen Anwendungen verdeutlicht: Im Jahr 2020 wurden in Deutschland ca. 3,2 Mio. Erythrozytenkonzentrate, 480.000 Thrombozytenkonzentrate, 630.000 Plasmakonserven sowie eine Vielzahl aus Spenden gewonnene Gerinnungs- und Immunglobulinpräparate [5] zur vitalen Therapie von Patienten eingesetzt. Die fachgerechte Anwendung dieser wertvollen lebensrettenden Produkte erfordert spezielles Wissen und Erfahrung aus mehreren Disziplinen der Medizin.

Die Interdisziplinäre Arbeitsgemeinschaft für klinische Hämotherapie (IAKH) – als Interessenvertretung der Transfusionsverantwortlichen, Transfusionsbeauftragten und Qualitätsbeauftragten Hämotherapie – begleitet und unterstützt die Kliniker nun schon seit 20 Jahren.

Von Beginn an widmet sich die IAKH der Fort‑, Weiterbildung und Unterstützung klinisch tätiger Ärzte

Am 16.02.2002 wurde auf den Marburger Transfusionsgesprächen mit der IAKH eine Arbeitsgemeinschaft gegründet [6], die durch die Verabschiedung des Transfusionsgesetzes (TFG) 1998 [2] und die Veröffentlichung der „Richtlinien zur Gewinnung von Blut und Blutbestandteilen und zur Anwendung von Blutprodukten (Hämotherapie)“ [7] im Juli 2000 notwendig geworden war. Dort waren Qualifikation und Aufgaben von Transfusionsverantwortlichen, Transfusionsbeauftragten, Qualitätsbeauftragten Hämotherapie und der Transfusionskommission beschrieben und mussten erstmalig verpflichtend für die Einrichtungen bestellt werden. Ihre Tätigkeit und Qualifikation muss jährlich erfasst und zusammen mit dem restlichen Status der Qualität bei der Anwendung von Blutprodukten der zuständigen Landesärztekammer gemeldet werden.

In dieser Situation widmete sich die IAKH gemäß Satzung vordringlich der Fortbildung und Weiterbildung der klinisch tätigen Ärzte und auch der Unterstützung bei aktuellen Fragen und Problemen in der Klinik. Sie richtete mehrere Veranstaltungen pro Jahr aus, die als „Marburger Transfusionsgespräche“ bekannt wurden. Treibende Kraft in den Anfangstagen war der in der klinischen Hämotherapie engagierte Internist, Transfusionsmediziner und Leiter des Instituts für Transfusionsmedizin und Hämostaseologie am Universitätsklinikum Marburg, Prof. Dr. med. Volker Kretschmer. Durch seine Beharrlichkeit und sein Engagement gelang es ihm damals schon im Schulterschluss mit der Landesärztekammer Hessen und vielen Anästhesisten eine hämotherapeutische Fachkompetenz bei vielen Klinikern herbeizuführen. Ein persönliches Hauptanliegen von Prof. Kretschmer war bereits vor der IAKH-Gründung 2002 die generelle Beratung aller Kliniker in transfusionsmedizinischen und hämostaseologischen Fragen.

An seiner Seite fanden sich von Anfang an Anästhesisten und Intensivmediziner. Sie haben Erfahrung mit der Anwendung von Blutkonserven – nicht nur bei herzchirurgischen oder sonstigen blutverbrauchenden Operationen. Durch die immense Patientennachfrage nach Eigenblut im Zuge des Skandals über Infektionen durch HIV-kontaminierte Blutprodukte der 1990er-Jahre hatten die Anästhesisten seit Jahren im Rahmen der Herstellung und des Betriebs von Eigenblutspendeeinrichtungen erheblichen transfusionsmedizinischen Sachverstand erworben. Unter allen Beteiligungen ist der fachdisziplinäre Beitrag der in der IAKH vertretenen Anästhesisten bis heute am größten. In der ersten Stunde waren mit Prof. Dr. med. Eberhard Götz aus Darmstadt, Dr. med. Arnulf Weiler-Lorentz aus Mannheim und Dr. med. Gerhard Wittenberg aus Ludwigshafen engagierte Anästhesisten als Mitstreiter im Boot. Das damalige Gründungsmitglied Prof. Dr. med. Rainer Moosdorf, der ehemalige herzchirurgische Chef an der Uniklinik Marburg, ist heute noch im aktiven Vorstand der IAKH. Beliebte und geschätzte Referenten auf den Transfusionsgesprächen der IAKH waren Anästhesisten wie Prof. Dr. med. Jürgen Biscoping vom St. Vincentius Krankenhaus Karlsruhe, der kürzlich verstorbene Prof. Dr. med. Hans Gombotz aus dem Universitätsklinikum Graz, Prof. Dr. med. Donat Spahn aus Zürich und Prof. Dr. med. Sibylle Kietaipl aus Wien.

Die Hilfestellung in der klinischen Tätigkeit durch die IAKH war und ist für viele bestellte Ärzte bis heute notwendig und wird dankbar angenommen. Die Blutspendedienste betrachten ihre Aufgabe als pharmazeutischer Hersteller mit der Abgabe der Blutkonserven als beendet. Die IAKH hilft bis heute dort, wo kein anderer sich zuständig fühlt, und nimmt ihren Platz in der medizinischen Fachgesellschaft ein. Durch die große Erfahrung vieler Anästhesisten mit autologen Techniken, die heute kaum mehr eine Rolle spielen, wie normovolämischer Hämodilution, kontrollierter Hypotension sowie Eigenblutspende unter Eisen- und Erythropoetineinsatz, wurden bereits sehr früh blutsparende Techniken im Sinne des heutigen „Patient Blood Management“ betrieben und praktiziert. Die maschinelle Autotransfusion ist nach wie vor ein Hauptaugenmerk der IAKH, wie die jüngste Metaanalyse zum Einsatz bei onkologischen Eingriffen zeigt [4]. Die führende Rolle der Anästhesisten beim Aufbau von PBM-Ambulanzen wird v. a. in mittleren und kleineren Häusern durch praktische Anleitungen, Empfehlungen und Ratschläge durch die IAKH gestützt.

Das satzungsgemäße Fördern der Qualität der Hämotherapie hat inzwischen viele Gesichter angenommen

In den letzten 20 Jahren hat sich die IAKH substanziell weiterentwickelt. Das satzungsgemäße Fördern der Qualität der Hämotherapie hat viele Gesichter angenommen, zeitgemäß auf der Internetseite der IAKH (www.iakh.de) zugänglich. Dort werden den Mitgliedern Musterformulare und Fortbildungen zur Verfügung gestellt, Kontakte angezeigt und im Forum Fragen beantwortet, die die Arbeit als Transfusionsverantwortliche, Transfusionsbeauftragte oder Qualitätsbeauftragte Hämotherapie erheblich erleichtern. Die IAKH ist im Arbeitskreis Blut vertreten, hat sich bei der Anhörung von Neufassungen der Richtlinie und der QuerschnittsLeitlinien der Bundesärztekammer eingebracht. Sie hat als Verfahrenseigner des Qualitätssicherungsverfahrens „Peer Review Hämotherapie“ den kollegialen Besuch zur Verbesserung der Hämotherapiepraxis erheblich geprägt und betreibt seit 2009 ein nationales kostenfreies Fehlerregister für die Anwendung von Blut- und Gerinnungsprodukten in Kooperation mit dem CIRSmedical der Bundesärztekammer. In unregelmäßigen Abständen bezieht die IAKH Stellung zu aktuellen Themen in der Hämotherapie wie z. B. der Vergütung und Freistellung der hämotherapeutischen Funktionsträger.

Neuere Initiativen sind die Ausschreibung und Verleihung eines Förderpreises, der Forschungsprojekte mit erheblicher Bedeutung für die klinische Hämotherapie unterstützt. Die Entwicklung einer software- und scannergestützten Absicherungstechnik bei der Anwendung von Blutkonserven und Plasmaderivaten durch die Arbeitsgruppe Transfusionssicherheit folgt der Zielsetzung eines der letzten Voten des Arbeitskreises Blut [1] zur Vermeidung von Fehltransfusionen und Verwechslungen. Eine computergestützte Simulationsschulung der Massivtransfusion in den anfordernden Kliniken hat pandemiebedingt noch zu wenig Auslastung erfahren. Für die meisten Ziele hat die IAKH Arbeitsgruppen gebildet. Weitere Initiativen umfassen die Mitarbeit im neuen Masterplan des Medizinstudiums, die Schließung einer faktischen Meldelücke für folgenlose Fehltransfusionen, die Entwicklung von Techniken zur Messbarkeit der Transfusionsindikation und vieles mehr.

Die IAKH wird auch weiterhin eine interdisziplinäre Plattform für Kliniker, Labor- und Transfusionsmediziner anbieten

Ganz entscheidend für die breite Akzeptanz der IAKH ist und bleibt die Interdisziplinarität nicht nur zwischen Anästhesisten sowie Transfusions- und Labormedizinern, sondern sind und bleiben auch die Kontakte zu Hämatologen, Onkologen, Kardiologen, Chirurgen sowie anderen klinischen Hämotherapeuten und deren Fachgesellschaften. In den letzten Jahren bestand auch überwiegend eine gute Zusammenarbeit mit den Landesärztekammern in ganz Deutschland und der Bundesärztekammer sowie dem Paul-Ehrlich-Institut. Intensive Partnerschaften konnten mit dem Arbeitsausschluss Bluttransfusion der Deutschen Gesellschaft für Anästhesiologie und Intensivmedizin (DGAI)/dem Berufsverband Deutscher Anästhesisten (BDA), der Deutschen Interdisziplinären Vereinigung für Intensiv- und Notfallmedizin (DIVI), der Gesellschaft für Thrombose- und Hämostaseforschung (GTH), dem Network for the Advancement of Patient Blood Management, Haemostasis and Thrombosis (NATA) und dem Aktionsbündnis Patientensicherheit (APS) eingegangen werden. Trotz guter und enger Zusammenarbeit mit den industriellen Sponsoren mit besonderer Bedeutung für die Hämotherapie wahrt die IAKH ihre Neutralität und Objektivität in Fach- und Präparatefragen.

Abschließend kann sich die deutsche Medizin beglückwünschen, mit der IAKH eine Institution verfügbar zu haben, die immer noch ehrenamtlich geführt und trotz eines geringen Mitgliedsbeitrags ein hohes und attraktives Leistungsangebot für den engagierten Hämotherapeuten hat. Die breite Anerkennung der Leistung von Hämotherapeuten ist ein noch nicht erreichtes Ziel der Gesellschaft. Bluttransfusion und Gerinnungstherapie als lebenswichtige Therapien wollen gut durch Fachkompetenz abgesichert sein, um sicherzustellen, dass die speziellen Produkte in geeigneter Dosis am richtigen Patienten zum richtigen Zeitpunkt angewandt werden. Wie sehr dies notwendig ist, zeigen die Berichte und Empfehlungen aus dem Fehlerregister der IAKH [3]. Die IAKH schaut anlässlich ihres Jubiläums stolz auf die 20 Jahre ihres Bestehens zurück und fühlt sich verpflichtet, weiterhin eine Plattform für den Dialog und die Fortbildung von Klinikern, Labormedizinern und Transfusionsmedizinern anzubieten.
